# Bergman projections on weighted Fock spaces in several complex variables

**DOI:** 10.1186/s13660-017-1560-3

**Published:** 2017-11-16

**Authors:** Xiaofen Lv

**Affiliations:** Department of Mathematics, Huzhou University, Huzhou, Zhejiang 313000 China

**Keywords:** Bergman kernel, Bergman projection, reverse-Hölder inequality

## Abstract

Let *ϕ* be a real-valued plurisubharmonic function on ${\mathbb {C}}^{n}$ whose complex Hessian has uniformly comparable eigenvalues, and let $\mathcal{F}^{p}(\phi)$ be the Fock space induced by *ϕ*. In this paper, we conclude that the Bergman projection is bounded from the *p*th Lebesgue space $L^{p}(\phi )$ to $\mathcal{F}^{p}(\phi)$ for $1\leq p \leq\infty$. As a remark, we claim that Bergman projections are also well defined and bounded on Fock spaces $\mathcal{F}^{p}(\phi)$ with $0< p<1$. We also obtain the estimates for the distance induced by *ϕ* and the $L^{p}(\phi)$-norm of Bergman kernel for $\mathcal{F}^{2}(\phi)$.

## Introduction

The symbol *dv* denotes the Lebesgue volume measure on ${\mathbb {C}}^{n}$, and

$$B(z,r)= \bigl\{ w\in{\mathbb {C}}^{n}: \vert w-z \vert < r \bigr\} \quad \text{for } z\in{\mathbb {C}}^{n} \text{ and } r>0. $$

Suppose $\phi: {\mathbb {C}}^{n}\rightarrow{\mathbb {R}}$ is a $C^{2}$ plurisubharmonic function. We say that *ϕ* belongs to the weight class **W** if *ϕ* satisfies the following statements:

(I) There exists $c> 0$ such that for $z\in{\mathbb {C}}^{n}$

1 $$ \inf_{z\in{\mathbb {C}}^{n}}\sup_{w\in B(z,c)}\Delta \phi(w)>0; $$

(II) Δ*ϕ* satisfies the reverse-Hölder inequality

2 $$ \Vert \Delta\phi \Vert _{L^{\infty}(B(z,r) )}\leq Cr^{-2n} \int_{B(z,r)}\Delta\phi \,dv, \quad\forall z\in{\mathbb {C}}^{n}, r > 0 $$

for some $0< C < +\infty$;

(III) The eigenvalues of $H_{\phi}$ are comparable, i.e., there exists $\delta_{0} > 0$ such that

$$ \bigl(H_{\phi}(z)u, u \bigr) \geq\delta_{0} \Delta\phi(z) \vert u \vert ^{2},\quad\forall z, u\in{\mathbb {C}}^{n}, $$

where

$$ H_{\phi}= \biggl(\frac{\partial^{2}\phi}{\partial z_{j}\,\partial\overline {z}_{k}} \biggr)_{j,k}. $$

Suppose $0< p<\infty$, $\phi\in\mathbf{W}$. The space $L^{p}(\phi)$ consists of all Lebesgue measurable functions *f* on ${\mathbb {C}}^{n}$ for which

$$\Vert f \Vert _{p,\phi}= \biggl( \int_{{\mathbb {C}}^{n}} \bigl\vert f(z) \bigr\vert ^{p}e^{-p\phi(z)} \,dv(z) \biggr)^{\frac {1}{p}}< \infty. $$

$L^{\infty}(\phi)$ is the set of all Lebesgue measurable functions *f* on ${\mathbb {C}}^{n}$ with

$$\Vert f \Vert _{\infty, \phi}=\sup_{z\in {\mathbb {C}}^{n}} \bigl\vert f(z) \bigr\vert e^{-\phi(z)} < \infty. $$

Let $H({\mathbb {C}}^{n})$ be the family of all holomorphic functions on ${\mathbb {C}}^{n}$. The weighted Fock space is defined as

$$\mathcal{F}^{p}(\phi)=L^{p}(\phi)\cap H\bigl({\mathbb {C}}^{n}\bigr) $$

with the same norm $\Vert \cdot \Vert _{p, \phi}$. It is easy to check that $\mathcal{F}^{p}(\phi)$ is a Banach space under $\Vert \cdot \Vert _{p, \phi}$ if $1\leq p<\infty $, and $\mathcal{F}^{p}(\phi)$ is a Fréchet space with the metric $\varrho(f,g)= \Vert f-g \Vert _{p, \phi}^{p}$ whenever $0< p<1$. Taking $\phi(z)=\frac{1}{2} \vert z \vert ^{2}$, $\mathcal {F}^{p}(\phi)$ is the classical Fock space which has been studied by many authors, see [1–3] and the references therein. Notice that the weight function *φ* on ${\mathbb {C}}^{n}$ with the restriction that $d d^{c} \varphi\simeq d d^{c} \vert z \vert ^{2}$ in [4] and [5] belongs to **W**.

In the one-dimensional case, an important contribution to weighted Fock spaces was given by Christ [6] (but see also [7, 8]). They work under the assumption that *ϕ* is subharmonic and that $\Delta\phi\, dA$ is a doubling measure, where *dA* is the area measure on ${\mathbb {C}}$. Notice that the hypotheses on $\Delta\phi\, dA$ are a sort of finite-type assumption and are automatically verified when *ϕ* is a subharmonic non-harmonic polynomial.

The result of Christ was extended by Delin to several complex variables under the assumption of strict plurisubharmonicity of the weight in [9]. Dall’Ara [10] tried to extend Christ’s approach to $n\geq2$. Given $\phi\in\mathbf{W}$, let $K(\cdot, \cdot)$ be the weighted Bergman kernel for $\mathcal{F}^{2}(\phi)$. In particular, Theorem 20 of [10] proves that there is a constant $C,\epsilon>0$ such that

3 $$ \bigl\vert K(z,w) \bigr\vert \leq C e^{\phi(z)+\phi(w)} \frac {e^{-\epsilon d(z,w)}}{\rho_{\phi}(z)^{n}\rho_{\phi}(w)^{n}} $$

for $z,w\in{\mathbb {C}}^{n}$, where $d(\cdot , \cdot)$, $\rho_{\phi }(\cdot)$ described in Section 2.

In the setting of Bergman spaces, the Bergman projection is bounded on *p*-Bergman spaces for $1< p<\infty$, it also maps $L^{\infty}$ into Bloch spaces, see [11] for details. With the Bergman kernel $K(\cdot, \cdot)$ for $\mathcal{F}^{2}(\phi)$, the Bergman projection *P* can be represented as

$$Pf(z)= \int_{{\mathbb {C}}^{n}}K(z,w)f(w)e^{-2\phi(w)}\,dv(w),\quad z\in {\mathbb {C}}^{n}. $$

It is well known that $P(f)=f$ for $f\in\mathcal{F}^{2}(\phi)$. The purpose of this work is to discuss the boundedness of Bergman projection acting on $\mathcal{F}^{p}(\phi)$ for general *p*. Section 2 is devoted to some basic estimates, including the distance $d(\cdot , \cdot)$ and the $L^{p}(\phi)$-norm of the Bergman kernel. In Section 3, we will discuss the boundedness of Bergman projections from $L^{p}(\phi)$ to $\mathcal{F}^{p}(\phi)$ with $1 \leq p \leq\infty$. We also show that the Bergman projection is well defined and bounded on $\mathcal{F}^{p}(\phi)$ for $p<1$.

In what follows, we always suppose $\phi\in\mathbf{W}$ and use *C* to denote positive constants whose values may change from line to line but do not depend on the functions being considered. Two quantities *A* and *B* are called equivalent, denoted by ‘$A\simeq B$’, if there exists some *C* such that $C^{-1 }A\le B \le C A$.

## Some basic estimates

In this section, we are going to give some estimates, which will be useful in the following section. At the beginning, we will give some notations.

For $z\in{\mathbb {C}}^{n}$, set

4 $$ \rho_{\phi}(z)=\sup \Bigl\{ r>0: \sup_{w\in B(z,r)} \Delta\phi (w)\leq r^{-2} \Bigr\} . $$

By (1), there exist $c, s > 0$ such that for $z\in{\mathbb {C}}^{n}$

$$ \sup_{w\in B(z, c)}\Delta\phi(w)\geq s. $$

We then have some $M>0$ such that

$$ \sup_{z\in{\mathbb {C}}^{n}}\rho_{\phi}(z)\leq M. $$

Moreover, there are some positive constants *C*, $M_{1}$ and $M_{2}$ such that for all $z,w\in{\mathbb {C}}^{n}$, we have

5 $$ C^{-1}\theta^{-M_{1}}\rho_{\phi}(w)\leq \rho_{\phi}(z)\leq C\theta ^{M_{2}}\rho_{\phi}(w), $$

where $\theta=\max (1,\frac{ \vert z-w \vert }{\rho _{\phi}(w)} )$. We can see this in Proposition 10 of [10].

Given $r>0$, write

$$B^{r}(z)=B\bigl(z,r\rho_{\phi}(z)\bigr) \quad \text{and} \quad B(z)=B^{1}(z). $$

Then (5) implies that there is some *C* such that for $z\in {\mathbb {C}}^{n}$

6 $$ C^{-1}\rho_{\phi}(w)\leq\rho_{\phi}(z) \leq C\rho_{\phi}(w)\quad \text{for }w\in B(z). $$

By (6) and the triangle inequality, we have $m_{1}, m_{2}>0$ such that

7 $$ B(z)\subseteq B^{m_{1}}(w), \qquad B(w)\subseteq B^{m_{2}}(z) \quad\text{whenever } w\in B(z). $$

Given a sequence $\{a_{k}\}_{k=1}^{\infty}$ in ${\mathbb {C}}^{n}$, we say that $\{a_{k}\}_{k=1}^{\infty}$ is a lattice if $\{B(a_{k}) \}_{k=1}^{\infty}$ covers ${{\mathbb {C}}^{n}}$ and the balls of $\{B^{\frac{1}{5}}(a_{k}) \}_{k=1}^{\infty}$ are pairwise disjoint. This lattice exists by a standard covering lemma, see Theorem 2.1 in [12], or Proposition 7 in [10] as well. Moreover, for the lattice $\{a_{k}\}_{k}$ and any $m> 0 $, there exists some integer *N* such that each $z\in{{\mathbb {C}}^{n}}$ can be in at most *N* balls of $\{B^{m}(a_{k}) \}_{k}$. Equivalently,

8 $$ \sum_{k=1}^{\infty}\chi_{B^{m}(a_{k})}(z)\le N \quad \text{for } z\in{{\mathbb {C}}^{n}}. $$

To the radius function $\rho_{\phi}$ defined as (4), we associate the Riemannian metric $\rho_{\phi}(z)^{-2}\,dz\otimes d\overline{z}$. In fact, we are interested only in the associated Riemannian distance, which we describe explicitly. If $\gamma: [0, 1]\rightarrow{\mathbb {C}}^{n}$ is piecewise $C^{1}$ curves, we define

$$ L_{\rho_{\phi}}(\gamma)= \int_{0}^{1}\frac{ \vert \gamma '(t) \vert }{\rho_{\phi}(\gamma(t))}\,dt. $$

Given $z, w \in{\mathbb {C}}^{n}$, we put

$$ d(z, w)=\inf_{\gamma}L_{\rho_{\phi}}(\gamma), $$

where the inf is taken as *γ* varies over the collection of curves with $\gamma(0)=z$ and $\gamma(1)=w$. We then have the estimate for this distance as follows.

### Lemma 1

*There exist* $\alpha,\beta, C>0$ *such that for* $z,w\in{\mathbb {C}}^{n}$

$$\frac{1}{C} \biggl(\frac{ \vert z-w \vert }{\rho_{\phi }(z)} \biggr)^{\alpha}\leq d(z,w)\leq C \biggl(\frac{ \vert z-w \vert }{\rho_{\phi}(z)} \biggr)^{\beta}. $$

### Proof

First, we claim that there is some $C>0$ such that

9 $$ d(z,w)\geq C \biggl(\frac{ \vert z-w \vert }{\rho_{\phi }(z)} \biggr)^{\alpha}. $$

In fact, set *μ* to be

10 $$ \mu\bigl(B(z,r)\bigr)=r^{2} \Vert \Delta\phi \Vert _{L^{\infty}(B(z,r))},\quad z\in{\mathbb {C}}^{n}, r>0. $$

By (2), it is easy to check that there is some $M>2$ such that

11 $$ \mu \bigl(B(z,2r) \bigr)\leq M\mu \bigl(B(z,r) \bigr). $$

Moreover,

12 $$ \mu \bigl(B\bigl(z,\rho_{\phi}(z)\bigr) \bigr)=1 $$

because of (4). Given any $r\leq R$, it is easy to check that

13 $$ \mu \bigl(B(z,r) \bigr)\leq \biggl(\frac{r}{R} \biggr)^{2}\mu \bigl(B(z,R) \bigr)\leq\mu \bigl(B(z,R) \bigr) $$

for $z\in{\mathbb {C}}^{n}$ because of (10). Also, there is a positive integer *m* such that $2^{m-1}r< R\leq2^{m}r$. Hence, (11) and (12) tell us

$$\mu \bigl(B(z,R) \bigr)\leq\mu \bigl(B\bigl(z,2^{m}r\bigr) \bigr)\leq M\mu \bigl(B\bigl(z,2^{m-1}r\bigr) \bigr)\leq\cdots\leq M^{m} \mu \bigl(B(z,r) \bigr). $$

Since $M^{m-1}=2^{(m-1)\log_{2}M}\leq (\frac{R}{r} )^{\log_{2}M}$, we get

14 $$ \mu \bigl(B(z,R) \bigr)\leq M \biggl(\frac{R}{r} \biggr)^{\log _{2}M}\mu \bigl(B(z,r) \bigr). $$

For $z,w\in{\mathbb {C}}^{n}$, notice that $B(w, \vert w-z \vert )\subset B(z,2 \vert w-z \vert )$. If $\vert w-z \vert < \rho_{\phi}(z)$, take any piecewise $C^{1}$ curve $\gamma:[0, 1]\rightarrow{\mathbb {C}}^{n}$ connecting *z* and *w*, and let $T_{0}$ be the minimum time such that $\vert z -\gamma(T_{0}) \vert =\rho_{\phi}(z)$. By (6), $\rho_{\phi}(\gamma(t))\simeq\rho_{\phi}(z)$ for $t\in[0,T_{0})$. This implies

$$\int_{0}^{1}\frac{ \vert \gamma'(t) \vert }{\rho_{\phi }(\gamma(t))}\,dt\geq \frac{C}{\rho_{\phi}(z)} \int_{0}^{T_{0}} \bigl\vert \gamma'(t) \bigr\vert \,dt\geq C\frac{ \vert z-w \vert }{\rho_{\phi}(z)}. $$

If $\vert z-w \vert \geq\rho_{\phi}(w)$, then (11), (10), (13) and (12) give

$$\begin{aligned} \mu \biggl(B \biggl(z,\frac{1}{4} \vert z-w \vert \biggr) \biggr)&\geq C\mu \bigl(B\bigl(z, 2 \vert z-w \vert \bigr) \bigr)\geq C\mu \bigl(B\bigl(w, \vert z-w \vert \bigr) \bigr) \\ &\geq C \biggl(\frac{ \vert z-w \vert }{\rho_{\phi }(w)} \biggr)^{2}\mu \bigl(B\bigl(w, \rho_{\phi}(w)\bigr) \bigr) \\ &=C \biggl(\frac{ \vert z-w \vert }{\rho_{\phi }(w)} \biggr)^{2}. \end{aligned}$$

On the other hand, for $\zeta\in\overline{B (z, \frac {1}{4} \vert z-w \vert )}$, there are

$$B \biggl(\zeta, \frac{1}{4} \vert z-w \vert \biggr)\subset B \biggl(z, \frac{1}{2} \vert z-w \vert \biggr) $$

and

$$B \biggl(z, \frac{1}{4} \vert z-w \vert \biggr)\subset B \biggl( \zeta, \frac{1}{2} \vert z-w \vert \biggr). $$

Combining the above with (11), we know

$$\mu \biggl(B \biggl(z,\frac{1}{4} \vert z-w \vert \biggr) \biggr) \simeq\mu \biggl(B \biggl(\zeta,\frac{1}{2} \vert z-w \vert \biggr) \biggr). $$

By the fact $\log_{2}M>0$, (13), (14) and (12), there exists $t>0$ such that

$$\begin{aligned} \mu \biggl(B \biggl(z,\frac{1}{4} \vert z-w \vert \biggr) \biggr) &\simeq\mu \biggl(B \biggl(\zeta,\frac{1}{2} \vert z-w \vert \biggr) \biggr) \\ &\leq C \biggl(\frac{ \vert z-w \vert }{\rho_{\phi }(\zeta)} \biggr)^{t}\mu \bigl(B \bigl( \zeta,\rho_{\phi}(\zeta ) \bigr) \bigr) \\ &\simeq \biggl(\frac{ \vert z-w \vert }{\rho_{\phi }(\zeta)} \biggr)^{t}. \end{aligned}$$

Hence, $(\frac{ \vert z-w \vert }{\rho_{\phi }(w)} )^{2}\leq C (\frac{ \vert z-w \vert }{\rho _{\phi}(\zeta)} )^{t}$. This implies

$$\rho_{\phi}(\zeta)\leq C \vert z-w \vert \biggl(\frac { \vert z-w \vert }{\rho_{\phi}(w)} \biggr)^{-\alpha },\qquad\zeta\in\overline{B \biggl(z, \frac{1}{4} \vert z-w \vert \biggr)}, $$

where $\alpha=\frac{2}{t}>0$. For any piecewise $C^{1}$ curves Γ, defined as $\gamma: [0,1]\rightarrow{\mathbb {C}}^{n}$ with $\gamma(0)=z$ and $\gamma(1)=w$, we have

$$\begin{aligned} \int_{\Gamma}\frac{ \vert \gamma'(t) \vert }{\rho _{\phi}(\gamma(t))}\,dt&\geq \int_{\Gamma\cap\overline{B (z, \frac{1}{4} \vert z-w \vert )}}\frac{ \vert \gamma'(t) \vert }{\rho_{\phi}(\gamma(t))}\,dt \\ &\geq\frac{1}{ \vert z-w \vert (\frac{ \vert z-w \vert }{\rho_{\phi}(w)} )^{-\alpha}} \int _{\Gamma\cap\overline{B (z, \frac{1}{4} \vert z-w \vert )}} \bigl\vert \gamma'(t) \bigr\vert \,dt \\ &\geq C \biggl(\frac{ \vert z-w \vert }{\rho_{\phi }(w)} \biggr)^{\alpha}. \end{aligned} $$

This yields (9) is true. Now, we are going to prove the other direction. For $z, w\in{\mathbb {C}}^{n}$, take $\gamma (t)=z+t(w-z)$ and $\gamma(t_{0})\in\partial B(z)$ (set $t_{0}=1$ if $w\in B(z)$). Then (5) gives

$$\begin{aligned} d(z,w)&\leq \vert w-z \vert \int_{0}^{1}\frac{dt}{\rho _{\phi}(\gamma(t))} \\ &\leq C \vert w-z \vert \biggl( \int_{0}^{t_{0}}+ \int _{t_{0}}^{1} \biggr)\frac{dt}{\rho_{\phi}(\gamma(t))} \\ &\leq C\frac{ \vert w-z \vert }{\rho_{\phi}(z)} \int_{0}^{1}dt+ C \biggl(\frac{ \vert w-z \vert }{\rho_{\phi}(z)} \biggr)^{1+M_{1}} \int_{0}^{1} t^{M_{1}}\,dt \\ &\leq C \biggl(\frac{ \vert w-z \vert }{\rho_{\phi }(z)} \biggr)^{\beta}, \end{aligned}$$

where $\beta>0$. The proof is completed. □

Now, we can estimate the following integral.

### Lemma 2

*Given* $p>0$ *and* $k\in\mathbb {R}$, *we have*

$$\int_{ {\mathbb {C}}^{n}}\rho_{\phi}(\zeta)^{k}e^{ - pd(z,\zeta )}\,dv( \zeta)\leq C\rho_{\phi}(z)^{k+2n}, $$

*where* $C>0$ *is a constant depending only on* *n*, *p* *and* *k*.

### Proof

By (6), it is easy to check that

$$\begin{aligned} \int_{B(z)}\rho_{\phi}(\zeta)^{k}e^{ - pd(z,\zeta)}\,dv( \zeta) \leq \int_{B(z)} \rho_{\phi}(\zeta)^{k}\,dv(\zeta) \leq C\rho_{\phi }(z)^{k+2n}. \end{aligned}$$

Estimate (9) gives

$$\begin{aligned} \int_{{{\mathbb {C}}^{n}}\backslash B(z)}\rho_{\phi}(\zeta)^{k}e^{ - pd(z,\zeta)}\,dv( \zeta) &\leq \int_{{{\mathbb {C}}^{n}}\backslash B(z)}\rho_{\phi}(\zeta)^{k}e^{ - pC_{1} (\frac{ \vert z-\zeta \vert }{\rho_{\phi }(z)} )^{\alpha}}\,dv( \zeta) \\ &\leq \int_{{{\mathbb {C}}^{n}}\backslash B(z)}\rho_{\phi}(\zeta )^{k} \int_{pC_{1} (\frac{ \vert z-\zeta \vert }{\rho _{\phi}(z)} )^{\alpha}}^{\infty}e^{-s}\,ds \,dv(\zeta) \\ &\leq \int_{pC_{1}}^{\infty}e^{-s} \int_{ B^{ (\frac {s}{pC_{1}} )^{\frac{1}{\alpha}}}(z)}\rho_{\phi}(\zeta )^{k}\,dv( \zeta)\,ds. \end{aligned}$$

By (5), the inequality above is no more than

$$\begin{aligned}& \int_{pC_{1}}^{\infty} \sup_{\zeta\in B^{ (\frac{s}{pC_{1}} )^{\frac {1}{\alpha}}}(z)} \rho_{\phi}(\zeta)^{k}v \bigl(B^{ (\frac {s}{pC_{1}} )^{\frac{1}{\alpha}}}(z) \bigr)e^{-s}\,ds \\& \quad \le C\rho_{\phi}(z)^{k+2n} \int_{pC_{1}}^{\infty}s^{\frac{2n+\max \{kM_{2}, -kM_{1}\}}{\alpha}}e^{-s}\,ds =C \rho_{\phi}(z)^{k+2n}. \end{aligned}$$

Therefore,

$$\int_{ {\mathbb {C}}^{n}}\rho_{\phi}(w)^{k}e^{ - pd(z,w) }\,dv(w) \leq C\rho_{\phi}(z)^{k+2n}. $$

The proof is completed. □

Next, we will give the $L^{p}(\phi)$-norm of the Bergman kernel $K(\cdot , \cdot)$ for $\mathcal{F}^{2}(\phi)$.

### Proposition 3

*For* $0< p<\infty$, *we have*

$$\bigl\Vert K(\cdot, z) \bigr\Vert _{p,\phi}\leq C e^{\phi(z)}\rho _{\phi}(z)^{2n (\frac{1}{p}-1 )}, \quad z\in{\mathbb {C}}^{n}. $$

### Proof

By (3) and Lemma 2, we obtain

$$\begin{aligned} \int_{{\mathbb {C}}^{n}} \bigl\vert K(w, z) \bigr\vert ^{p}e^{-p\phi(w)}\,dv(w) &\le C\frac{e^{p\phi(z)}}{\rho_{\phi}(z)^{pn}} \int_{{\mathbb {C}}^{n}}\rho_{\phi}(w)^{-pn}e^{-p\epsilon d(z,w)}\,dv(w) \\ &\leq Ce^{p\phi(z)}\rho_{\phi}(z)^{2n(1-p)}. \end{aligned}$$

The proof is completed. □

### Lemma 4

*For* $0< p<\infty$, *there is a constant* $C>0$ *such that for all* $r\in (0,1]$, $f\in H({\mathbb {C}}^{n})$ *and* $z\in{\mathbb {C}}^{n}$, *we have*

15 $$ \bigl\vert f(z) \bigr\vert e^{-\phi(z)}\leq \frac{C}{r^{\frac {2n}{p}}\rho_{\phi}(z)^{\frac{2n}{p}}} \biggl( \int_{ B^{r}(z)} \bigl\vert f(w)e^{-\phi(w)} \bigr\vert ^{p}\,dv(w) \biggr)^{\frac{1}{p}}. $$

### Proof

If $p=2$, (15) is just Lemma 13 in [10]. For $p\neq2$, we borrow the idea in Lemma 19 of [7] and Lemma 13 in [10]. The details are omitted. □

## Boundedness of Bergman projections

Recall that the Bergman projection *P* on $L^{p}(\phi)$ is defined as

$$Pf(z)= \int_{{\mathbb {C}}^{n}}K(z,w)f(w)e^{-2\phi(w)}\,dv(w),\quad z\in {\mathbb {C}}^{n}. $$

In this section, we focus on the boundedness of Bergman projections *P* from $L^{p}(\phi)$ to $\mathcal{F}^{p}(\phi)$ for $1\leq p\leq \infty$.

### Theorem 5

*Let* $1\leq p\leq\infty$. *Then the Bergman projection* *P* *is bounded as a map from* $L^{p}(\phi)$ *to* $\mathcal{F}^{p}(\phi)$.

### Proof

By the definition of *P*, we can conclude *Pf* is holomorphic on ${\mathbb {C}}^{n}$. Fubini’s theorem and Proposition 3 yield

$$\begin{aligned} \Vert Pf \Vert _{1,\phi} &\leq \int_{{\mathbb {C}}^{n}}e^{-\phi(z)}\,dv(z) \int_{{\mathbb {C}}^{n}} \bigl\vert K(z,w)f(w) \bigr\vert e^{-2\phi(w)}\,dv(w) \\ &= \int_{{\mathbb {C}}^{n}} \bigl\vert f(w) \bigr\vert e^{-2\phi (w)}\,dv(w) \int_{{\mathbb {C}}^{n}} \bigl\vert K(z,w) \bigr\vert e^{-\phi(z)}\,dv(z) \\ &\leq C \Vert f \Vert _{1,\phi} \end{aligned}$$

for $f\in L^{1}(\phi)$. Given $f\in L^{\infty}(\phi)$, we obtain

$$\begin{aligned} \Vert Pf \Vert _{\infty,\phi} &\leq\sup_{z\in{\mathbb {C}}^{n}}e^{-\phi(z)} \int_{{\mathbb {C}}^{n}} \bigl\vert K(z,w)f(w) \bigr\vert e^{-2\phi(w)}\,dv(w) \\ &\leq \Vert f \Vert _{\infty,\phi}\sup_{z\in{\mathbb {C}}^{n}}e^{-\phi(z)} \int_{{\mathbb {C}}^{n}} \bigl\vert K(z,w) \bigr\vert e^{-\phi(w)}\,dv(w) \\ &\leq C \Vert f \Vert _{\infty,\phi}. \end{aligned}$$

If $1< p<\infty$, Hölder’s inequality and Fubini’s theorem give

$$\begin{aligned}& \Vert Pf \Vert ^{p}_{p,\phi} \\& \quad \leq \int_{{\mathbb {C}}^{n}}e^{-p\phi(z)}\,dv(z) \biggl( \int_{{\mathbb {C}}^{n}} \bigl\vert K(z,w)f(w) \bigr\vert e^{-2\phi(w)}\,dv(w) \biggr)^{p} \\& \quad \leq \int_{{\mathbb {C}}^{n}} \int_{{\mathbb {C}}^{n}} \bigl\vert f(w) \bigr\vert ^{p}e^{-p\phi(w)} \bigl\vert K(z,w) \bigr\vert e^{-\phi(w)}\,dv(w) \bigl\Vert K(z,\cdot) \bigr\Vert _{1,\phi}^{p-1}e^{-p\phi(z)}\,dv(z) \\& \quad \leq C \int_{{\mathbb {C}}^{n}}e^{-\phi(z)}\,dv(z) \int_{{\mathbb {C}}^{n}} \bigl\vert f(w) \bigr\vert ^{p}e^{-p\phi(w)} \bigl\vert K(z,w) \bigr\vert e^{-\phi(w)}\,dv(w) \\& \quad \leq C \int_{{\mathbb {C}}^{n}} \bigl\vert f(w) \bigr\vert ^{p}e^{-p\phi (w)}e^{-\phi(w)}\,dv(w) \int_{{\mathbb {C}}^{n}} \bigl\vert K(z,w) \bigr\vert e^{-\phi(z)}\,dv(z) \\& \quad \leq C \Vert f \Vert ^{p}_{p,\phi} \end{aligned}$$

for $f\in L^{p}(\phi)$. Thus, *P* is bounded from $L^{p}(\phi)$ to $\mathcal{F}^{p}(\phi)$ for $1\leq p\leq\infty$. The proof is ended. □

In addition, we observe that the Bergman projection is also well defined and bounded on the weighted Fock space $\mathcal{F}^{p}(\phi )$ with $p<1$.

### Remark 6

For $p<1$, the Bergman projection *P* is bounded on $\mathcal {F}^{p}(\phi)$.

### Proof

First, we claim that *P* is well defined on $\mathcal {F}^{p}(\phi)$. In fact, given any $f\in\mathcal{F}^{p}(\phi)$, by (3), (15) and Lemma 2, we obtain

$$\begin{aligned}& \begin{gathered} \int_{{\mathbb {C}}^{n}} \bigl\vert K(z,w)f(w) \bigr\vert e^{-2\phi(w)}\,dv(w) \\ \quad \leq C \Vert f \Vert _{p,\phi} \int_{{\mathbb {C}}^{n}}\rho _{\phi}(w)^{-\frac{2n}{p}} \bigl\vert K(z,w) \bigr\vert e^{-\phi(w)}\,dv(w) \end{gathered} \\& \begin{gathered} \quad \leq Ce^{\phi(z)}\rho_{\phi}(z)^{-n} \int_{{\mathbb {C}}^{n}}\rho _{\phi}(w)^{-\frac{2n}{p}-n}e^{ - \epsilon d(z,w)}\,dv(w) \\ \quad \leq Ce^{\phi(z)}\rho_{\phi}(z)^{-\frac{2n}{p}}< \infty. \end{gathered} \end{aligned}$$

Now, we deal with the boundedness of *P*. In fact, let $\{a_{k}\}_{k}$ be the lattice. For $f\in\mathcal {F}^{p}(\phi)$, we get

$$\begin{aligned} \bigl\vert P f(z) \bigr\vert ^{p}&\leq \Biggl(\sum _{k=1}^{\infty} \int_{B(a_{k})} \bigl\vert f(w)K(w,z) \bigr\vert e^{-2\phi(w)}\,dv(w) \Biggr)^{p} \\ &\leq\sum_{k=1}^{\infty} \biggl( \int_{B(a_{k})} \bigl\vert f(w)K(w,z) \bigr\vert e^{-2\phi(w)}\,dv(w) \biggr)^{p} \\ &\leq\sum_{k=1}^{\infty}v \bigl(B(a_{k}) \bigr)^{p} \Bigl(\sup_{w\in B(a_{k})} \bigl\vert f(w)K(w,z) \bigr\vert e^{-2\phi(w)} \Bigr)^{p}. \end{aligned}$$

Notice that the associated function $\rho_{2\phi}=\frac{\sqrt {2}}{2}\rho_{\phi}$, which follows from (4). Applying Lemma 4 with weight 2*ϕ* instead of *ϕ*, there then is some constant $C>0$ such that $\vert P f(z) \vert ^{p}$ is no more than *C* times

$$\begin{aligned} \sum_{k=1}^{\infty}\rho_{\phi }(a_{k})^{2np-2n} \sup_{w\in B(a_{k})} \int_{B(w)} \bigl\vert f(u) \bigr\vert ^{p} \bigl\vert K(u,z) \bigr\vert ^{p} e^{-2p\phi(u)}\,dv(u). \end{aligned}$$

Combining (7) with (8), we obtain

$$\begin{aligned} \bigl\vert P f(z) \bigr\vert ^{p} &\leq C\sum _{k=1}^{\infty} \int_{B^{m_{2}}(a_{k})}\rho_{\phi }(u)^{2np-2n} \bigl\vert f(u) \bigr\vert ^{p} \bigl\vert K(u,z) \bigr\vert ^{p} e^{-2p\phi(u)}\,dv(u) \\ &\leq C N \int_{{\mathbb {C}}^{n}}\rho_{\phi}(u)^{2np-2n} \bigl\vert f(u) \bigr\vert ^{p} \bigl\vert K(u,z) \bigr\vert ^{p} e^{-2p\phi(u)}\,dv(u). \end{aligned}$$

Therefore, applying Fubini’s theorem and Proposition 3, we get

$$\begin{aligned}& \int_{{\mathbb {C}}^{n}} \bigl\vert P f(z) \bigr\vert ^{p}e^{-p\phi (z)}\,dv(z) \\& \quad \leq C \int_{{\mathbb {C}}^{n}} \int_{{\mathbb {C}}^{n}} \bigl\vert K(u,z) \bigr\vert ^{p} e^{-p\phi(z)}\,dv(z)\rho_{\phi}(u)^{2np-2n} \bigl\vert f(u) \bigr\vert ^{p} e^{-2p\phi(u)}\,dv(u) \\& \quad \leq C \int_{{\mathbb {C}}^{n}} \bigl\vert f(u) \bigr\vert ^{p} e^{-p\phi (u)}\,dv(u). \end{aligned}$$

This means that *P* is bounded on $\mathcal{F}^{p}(\phi)$. The proof is ended. □

## Conclusion

In this paper, we show the boundedness of Bergman projection from the *p*th Lebesgue space $L^{p}(\phi)$ to the weighted Fock space $\mathcal {F}^{p}(\phi)$ for $1\leq p\leq\infty$. We also remak that the Bergman projection is bounded on $\mathcal{F}^{p}(\phi)$ with $p<1$. Meanwhile, we get the estimates for the distance induced by *ϕ* and the $L^{p}(\phi)$-norm of Bergman kernel for $\mathcal{F}^{2}(\phi )$.

## References

[1] Hu ZJ, Lv XF (2011). Toeplitz operators from one Fock space to another. Integral Equ. Oper. Theory.

[2] Isralowitz J, Zhu KH (2010). Toeplitz operators on the Fock space. Integral Equ. Oper. Theory.

[3] Zhu KH (2012). Analysis on Fock Spaces.

[4] Hu ZJ, Lv XF (2014). Toeplitz operators on Fock spaces $F^{p}(\varphi)$. Integral Equ. Oper. Theory.

[5] Schuster AP, Varolin D (2012). Toeplitz operators and Carleson measures on generalized Bargmann-Fock spaces. Integral Equ. Oper. Theory.

[6] Christ M (1991). On the *∂̅* equation in weighted $L^{2}$ norms in $\mathbb {C}$. J. Geom. Anal..

[7] Marco N, Massanedv X, Ortega-Cerdà J (2003). Interpolating and sampling sequences for entire functions. Geom. Funct. Anal..

[8] Marzo J, Ortega-Cerdà J (2009). Pointwise estimates for the Bergman kernel of the weighted Fock space. J. Geom. Anal..

[9] Delin H (1998). Pointwise estimates for the weighted Bergman projection kernel in ${\mathbb {C}}^{n}$, using a weighted $L^{2}$ estimate for the *∂̅* equation. Ann. Inst. Fourier (Grenoble).

[10] Dall’Ara GM (2015). Pointwise estimates of weighted Bergman kernels in several complex variables. Adv. Math..

[11] Hedenmalm H, Korenblum B, Zhu KH (2000). Theory of Bergman Spaces.

[12] Mattila P (1995). Geometry of Sets and Measures in Euclidean Spaces, Fractals and Rectifiability.

